# Perinatal Bisphenol A Exposure and Reprogramming of Imprinted Gene Expression in the Adult Mouse Brain

**DOI:** 10.3389/fgene.2019.00951

**Published:** 2019-10-10

**Authors:** Maureen A. Malloy, Joseph J. Kochmanski, Tamara R. Jones, Justin A. Colacino, Jaclyn M. Goodrich, Dana C. Dolinoy, Laurie K. Svoboda

**Affiliations:** ^1^Department of Environmental Health Sciences, University of Michigan School of Public Health, Ann Arbor, MI, United States; ^2^Department of Translational Science & Molecular Medicine, College of Human Medicine, Michigan State University, Grand Rapids, MI, United States; ^3^Department of Nutritional Sciences, University of Michigan School of Public Health, Ann Arbor, MI, United States

**Keywords:** bisphenol A, imprinted genes, DNA methylation, DNA hydroxymethylation, brain

## Abstract

Genomic imprinting, a phenomenon by which genes are expressed in a monoallelic, parent-of-origin-dependent fashion, is critical for normal brain development. Expression of imprinted genes is regulated *via* epigenetic mechanisms, including DNA methylation (5-methylcytosine, 5mC), and disruptions in imprinting can lead to disease. Early-life exposure to the endocrine disrupting chemical bisphenol A (BPA) is associated with abnormalities in brain development and behavior, as well as with disruptions in epigenetic patterning, including 5mC and DNA hydroxymethylation (5-hydroxymethylcytosine, 5hmC). Using an established mouse model of perinatal environmental exposure, the objective of this study was to examine the effects of perinatal BPA exposure on epigenetic regulation of imprinted gene expression in adult mice. Two weeks prior to mating, dams were assigned to control chow or chow containing an environmentally relevant dose (50 µg/kg) of BPA. Exposure continued until offspring were weaned at post-natal day 21, and animals were followed until 10 months of age. Expression of three imprinted genes—*Pde10a, Ppp1r9a*, and *Kcnq1*, as well as three genes encoding proteins critical for regulation of 5mC and 5hmC—*Dnmt1*, *Tet1*, and *Tet2*, were evaluated in the right cortex and midbrain using qRT-PCR. Perinatal BPA exposure was associated with a significant increase in adult *Kcnq1* (p = 0.04) and *Dnmt1* (p = 0.02) expression in the right cortex, as well as increased expression of *Tet2* in the midbrain (p = 0.03). Expression of *Tet2* and *Kcnq1* were positively correlated in the midbrain. Analysis of 5mC and 5hmC at the *Kcnq1* locus was conducted in parallel samples using standard and oxidative bisulfite conversion followed by pyrosequencing. This analysis revealed enrichment of both 5mC and 5hmC at this locus in both brain regions. No significant changes in 5mC and 5hmC at *Kcnq1* were observed with perinatal BPA exposure. Together, these data suggest that perinatal BPA exposure results in altered expression of *Kcnq1*, *Dnmt1*, and *Tet2* in the adult mouse brain. Further studies with larger sample sizes are necessary to understand the mechanistic basis for these changes, as well as to determine the implications they have for brain development and function.

## Introduction

The Developmental Origins of Health and Disease (DOHaD) hypothesis states that environmental exposures during critical windows of development influence the risk of diseases later in life (Barker, 2007). An important mechanism by which developmental exposures can affect long-term disease risk is *via* disruption of the normal epigenetic processes regulating gene expression (Dolinoy and Jirtle, 2008). One critical epigenetic mechanism of gene regulation is DNA methylation, the addition of a methyl group to the fifth position of cytosines in cytosine-phosphate-guanine (CpG) dinucleotides (5-methylcytosine, 5mC) (Jones, 2012). DNA methylation is critical for establishment of tissue-specific gene expression patterns, regulation of imprinted genes, maintenance of genome stability, and silencing of transposable elements (Jones, 2012). Because it is stable, mitotically heritable, and undergoes rapid reprogramming during early development, DNA methylation is particularly vulnerable to the effects of toxicant exposures (Dolinoy and Jirtle, 2008). 5mC can be further oxidized to 5-hydroxymethylcytosine (5hmC) (Lopez et al., 2017). 5hmC was initially proposed to be an intermediate in the DNA demethylation pathway; however, recent studies suggest that the genomic distribution, downstream targets, and functional roles of 5hmC are distinct from that of 5mC (Lopez et al., 2017). 5mC and 5hmC undergo dynamic changes during brain development, and both marks play a critical role in neuronal function, including learning and memory, during development and adulthood (Spiers et al., 2015; Jobe and Zhao, 2017; Spiers et al., 2017). Alterations in these marks are associated with neurodegenerative and psychiatric illnesses, suggesting a role for 5mC and 5hmC in disease pathogenesis (Zhao et al., 2017; Cui and Xu, 2018).

Genomic imprinting is an epigenetic mechanism resulting in mono-allelic, parent-of-origin expression of a subset of genes. Genomic imprinting is mediated in large part by DNA methylation, in cooperation with histone marks and long non-coding RNAs (Reik and Walter, 2001; Barlow and Bartolomei, 2014). During primordial germ cell development, the parental imprinting marks are erased, and new imprinting marks are then re-established, in a pattern consistent with the sex of the developing embryo (Reik and Walter, 2001). Because imprinted genes are functionally haploid, environmental exposures may be more likely to disrupt their expression and function, with potentially serious health consequences (Dolinoy et al., 2007). We and others have recently reported that perinatal environmental exposures disrupt programming of 5mC and 5hmC at imprinted loci in pancreatic islets (Susiarjo et al., 2013), blood (Li et al., 2016; Kochmanski et al., 2018), and mouse tail samples (Faulk et al., 2014), and lead to alterations in imprinted gene expression (Susiarjo et al., 2013; Kochmanski et al., 2018). Proper expression of imprinted genes is critical for normal neurodevelopment, and disruptions in imprinting are associated with several human brain disorders (Perez et al., 2016).

Bisphenol A (BPA) is a widely used, endocrine disrupting chemical found in a variety of consumer products, including food containers, receipt paper, and medical equipment. Widespread use of BPA has resulted in ubiquitous human exposure (Vandenberg et al., 2007; Vandenberg et al., 2010). Perinatal exposure to BPA is associated with a number of adverse neurodevelopmental, behavioral, and psychiatric outcomes in human and animal models, including attention deficit/hyperactivity behaviors, learning deficits, anxiety, and depression (Matsuda et al., 2012; Harley et al., 2013; Johnson et al., 2016; Xin et al., 2018). Furthermore, recent work suggests that perinatal BPA exposure may lead to altered expression of imprinted genes in the offspring brain (Drobna et al., 2018), and that BPA-induced alterations in DNA methylation may play a role in BPA-mediated adverse effects on brain development (Kundakovic et al., 2013; Kumar and Thakur, 2017; Drobna et al., 2018). Based on this evidence, we hypothesized that perinatal BPA exposure would result in altered epigenetic programming of imprinted gene expression in the brain, long after cessation of exposure. In particular, given our recent finding that perinatal BPA exposure alters DNA methylation and hydroxymethylation at imprinted loci in the blood (Kochmanski et al., 2018), we hypothesized that these changes in imprinted gene expression would be mediated by programming of 5hmC and/or 5mC. To address these questions, we utilized an established mouse model of perinatal BPA exposure to investigate whether BPA alters programming of imprinted gene expression at 10 months of age. For this study, we investigated three imprinted genes: *Kcnq1*, *Ppp1r9a*, and *Pde10a*. We previously found that these genes were differentially hydroxymethylated in the blood of mice after perinatal exposure to BPA (Kochmanski et al., 2018). Moreover, mutations and/or disruption in expression of these genes are associated with neurological and psychiatric illnesses (Goldman et al., 2009; Konopaske et al., 2015; Kelly, 2018).

## Methods

### Exposure Paradigm and Brain Collection

**Figure 1:**
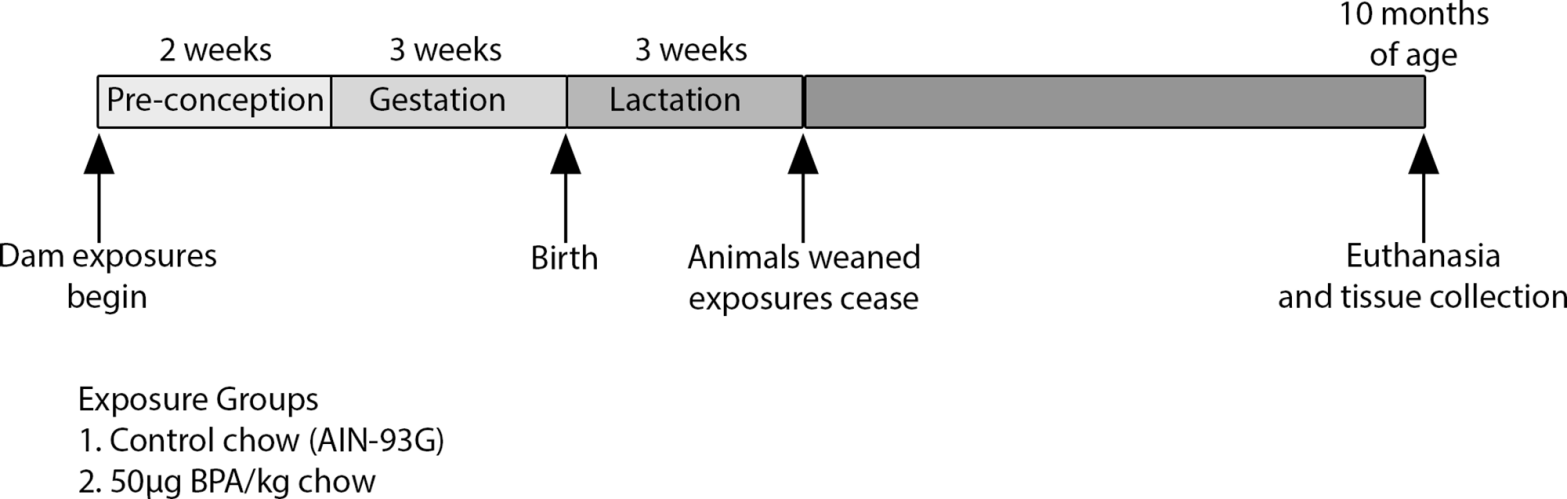
Exposure paradigm and experimental timeline. Dams were exposed to control or BPA-containing chow starting two weeks prior to mating until weaning. Exposures ceased on offspring postnatal day 21, and animals were administered control chow until 10 months of age. Offspring brains were collected and sectioned at 10 months of age. Six mice (three males and three females) per group were analyzed in this study.

Mice utilized for breeding and exposure were obtained from a colony maintained for over 230 generations with the A^vy^ (viable yellow agouti) allele passed through the male line, resulting in forced heterozygosity on a genetically invariant background with 93% identity to C57BL/6J (Waterland and Jirtle, 2003; Weinhouse et al., 2014). Two weeks prior to mating with A^vy^/a males, eight to ten-week-old virgin *a/a* dams were assigned to experimental diets consisting of control AIN-93G chow or chow containing 50 µg BPA/kg. Chow was supplied by Harlan Teklad, and BPA was supplied by the NTP (National Toxicology Program, Durham NC). The dose of BPA was chosen based on published data demonstrating that this results in human-relevant exposure levels (Anderson et al., 2012). Dams were kept on experimental diets *ad libitum* beginning 2 weeks prior to mating through pregnancy and lactation. On postnatal day 21, exposures ceased and offspring were weaned to control AIN-93G diet and followed until 10 months of age. Only wild-type, a/a offspring were followed for this study. At 10 months of age, mice were euthanized and the right cortex, left cortex, and midbrain of each animal were dissected out, flash-frozen and stored at -80°C for later use. This study utilized right cortex and midbrain samples (n = 6 control and 6 BPA-exposed) from the same a/a offspring in which BPA-induced changes in 5mC and 5hmC were identified in the blood (Kochmanski et al., 2018). Each exposure group had three males and three females. **Figure 1** provides a summary of the exposure paradigm and experimental design. All animal studies were carried out in an AAALAC accredited facility, under approval by the University of Michigan Institutional Animal Care & Use Committee (IACUC) and in compliance with the Guide for the Care and Use of Laboratory Animals.

### DNA and RNA Isolation

DNA and RNA were isolated from brain samples using the Qiagen Allprep DNA/RNA/miRNA Universal Kit (Qiagen, Cat. #80224). Briefly, frozen samples from the right cortex and midbrain of each mouse were thawed to the point where 30 mg could be excised from each brain section. Tissue samples were then homogenized using a TissueLyser (Qiagen) and treated according to the Qiagen Allprep DNA/RNA/miRNA Universal Kit Protocol. All isolated RNA and DNA samples were assessed for yield and purity using a NanoDrop spectrophotometer.

### Analysis of Gene Expression Using Quantitative Real-Time PCR

Expression of three imprinted genes previously identified to be differentially hydroxymethylated with perinatal BPA exposure (Kochmanski et al., 2018)—*Kcnq1*, *Pde10a*, and *Ppp1r9a* and three genes encoding enzymes that catalyze the formation of 5mC and 5hmC—*Dnmt1*, *Tet1* and *Tet2* were quantified separately by brain region for each of the twelve mice using quantitative real-time PCR (qRT-PCR). qRT-PCR primers were designed using the online Genscript Real-time PCR Primer Design software, and specificity for all designed primers was checked using the NCBI Primer-BLAST online tool. See **Supplementary Table 1** for primer sequences; 1000 ng of RNA from each sample was reverse transcribed into complementary DNA (cDNA) using the Bio-Rad iScript cDNA Synthesis Kit (Cat. #1708890). cDNA was diluted 1:5 in RNase-free water, mixed with 10 µM forward/reverse primers, nuclease-free water, and Bio-Rad iQ SYBR Green Supermix (Cat. #1708880). qRT-PCR was performed using the pre-programmed 2-step PCR+ melt curve protocol on a Bio-rad CFX96 Real-Time System C1000 Thermal Cycler (Bio-Rad; Hercules, CA). All target genes and three housekeeping genes (β-actin, *18S*, *GAPDH*) for each brain sample were run in triplicate on the same plate. Samples in which the coefficient of variation between the triplicates was greater than 10% were discarded and rerun. Data were normalized to the average Ct value for all three housekeeping genes. Relative gene expression was calculated separately by brain region using the ^ΔΔ^Ct method (Yuan et al., 2006; Rao et al., 2013). Statistical analysis was performed using a two-sided non-parametric Wilcoxon test using the *stats* package in R. Summary statistics were obtained using the *EpiDisplay* package in R. Graphs were generated using the *ggplot2* package in R.

### DNA Methylation and Hydroxymethylation Quantification

Pyrosequencing primers for *Kcnq1* were designed using the PyroMark Assay Design Software 2.0 Methylation Analysis (CpG) Assay and the July 2007 NCBI37/mm9 mouse genome (**Supplementary Table 2**). Primers were designed to target a CpG within an intronic region reported to be differentially hydroxymethylated with perinatal BPA exposure in the blood (Kochmanski et al., 2018); see **Figure 4** for location. Primers were assessed for mispriming using the BiSearch: Primer Design and Search Tool. DNA samples and low and high mouse DNA methylation standards from EpigenDX were bisulfite and oxidative bisulfite treated according to the NuGEN TrueMethyl oxBS Module protocol (Cat. #0414-32). Briefly, 1 µg of input genomic DNA was adjusted with nuclease-free water to 50 µl and each genomic DNA sample was divided into two aliquots. Each aliquot underwent independent, parallel treatments and were either oxidative bisulfite converted or mock oxidative bisulfite converted according to the TrueMethyl protocol. The yield and purity of treated samples were quantified using a NanoDrop spectrophotometer. The target locus within *Kcnq1* was PCR amplified in both the bisulfite and oxidative bisulfite converted samples. PCR products were verified using the QIAxcel automated DNA electrophoresis. DNA methylation levels were quantified using the PyroMark Q96 ID instrument (Qiagen). All plates were run with bisulfite and oxidative bisulfite converted low and high DNA methylation standards from EpigenDX. Samples that failed the PyroMark internal quality control assessment were rerun and only methylation percentages that passed were used for analysis. Bisulfite converted samples reveal the total level of 5mC + 5hmC, while oxidative bisulfite treated samples show total levels of 5mC. Thus, 5hmC levels were quantified by subtracting the result obtained from the oxidative bisulfite converted sample (5mC) from the bisulfite converted sample (5mC + 5hmC). For statistical analysis, Mann-Whitney and mixed linear effects models were used to asses differences in methylation and hydroxymethylation by exposure group. Analyses in the right cortex and midbrain were conducted separately. Exposure group, sex and methylation category (5mC or 5hmC) were included in all models, and a random factor for sample ID was included in all models to account for sample autocorrelation from matched 5mC and 5hmC percentages for each individual. The *lme4* package within the statistical program R was used for these analyses. Raw pyrosequencing data can be found in **Supplementary Table 4**. Correlation analyses were assessed with a Spearman Correlation Test using the *ggpubr* package in R. Correlation plots depict Ct values that have been normalized to the average Ct value of the three housekeeping genes.

## Results

### Litter Parameters and Phenotypic Data

To investigate the effects of BPA exposure on epigenetic regulation of imprinted gene expression, we utilized brain tissue from a previous study in an established mouse model of perinatal BPA exposure (Kochmanski et al., 2018) (see *Methods*). Dams were exposed to BPA or control chow beginning two weeks prior to mating until weaning at post-natal day (PND) 21 (**Figure 1**). Thus, offspring were exposed to BPA during pregnancy and lactation. After PND21, exposures ceased and all animals received control chow until euthanasia at 10 months of age. Developmental BPA exposure had no effect on litter size, ratio of males to females, or the a/a to Avy/a genotypic ratio (Kochmanski et al., 2018). As noted previously, BPA exposure resulted in a significant reduction in the number of pups per litter that survived at weaning (Kochmanski et al., 2017).

### Perinatal BPA Exposure Alters Imprinted Gene Expression in the Mouse Brain

**Figure 2:**
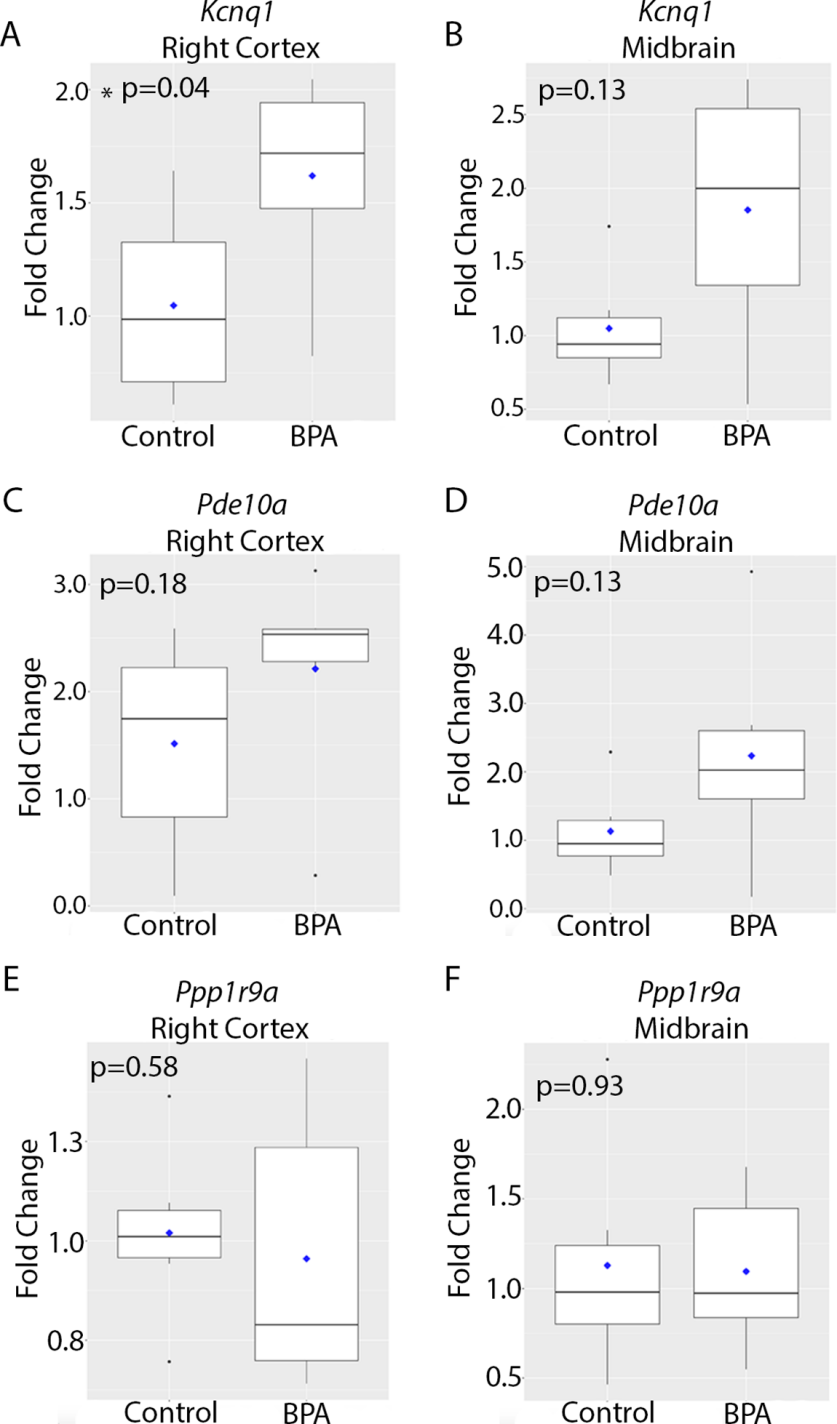
Imprinted gene expression in the brain at 10 months of age in animals exposed to control vs. BPA. Box plots depicting qRT-PCR data for *Kcnq1* **(A**, **B)**, *Pde10a* **(C**, **D)**, and *Pp1r9a* **(E**, **F)** in the right cortex **(A**, **C**, **E)** and midbrain **(B**, **D**, **F)**. Data were analyzed using a two-sided non-parametric Wilcoxon test. Graphs depict the fold change in gene expression relative to the average of the control samples. n = 6 mice per exposure.

To assess the effects of perinatal BPA exposure on expression of imprinted genes, we used qRT-PCR to measure expression of *Kcnq1*, *Pde10a*, and *Ppp1r9a* in the brains of 10-month-old mice. Notably, although BPA exposure was discontinued at three weeks of age, *Kcnq1* expression in the right cortex was significantly higher in BPA-exposed animals compared to controls at 10 months of age (p = 0.04) (**Figure 2A** and **Table 1**). In the midbrain, there was a trend toward increased expression of *Kcnq1* in BPA-exposed animals that did not reach statistical significance (p = 0.13) (**Figure 2B** and **Table 1**). *Pde10a* expression was higher in the BPA exposed group compared to the control group in both the right cortex and midbrain, although the differences did not reach statistical significance (p = 0.18 and 0.13 for right cortex and midbrain respectively, **Figures 2C, D** and **Table 1**). *Ppp1r9a* expression in the right cortex and midbrain did not differ between control and BPA-exposed mice (**Figures 2E, F** and **Table 1**). Collectively, these data demonstrate that perinatal BPA exposure altered *Kcnq1* expression in the brains of adult mice, months after discontinuation of exposure.

**Table 1:** *Kcnq1* qRT-PCR Summary Statistics.

Gene	Region	Exposure	N	Mean^a^ (SD)	Median^a^ (Range)	Mann-Whitney test (α = 0.05)
*Kcnq1*	Right Cortex	Control	6	16.18 (0.60)	16.13 (15.46-16.89)	0.04
		BPA	6	15.54 (0.49)	15.40 (15.14-16.46)	
	Midbrain	Control	6	16.43 (0.47)	16.51 (15.63-17.01)	0.13
		BPA	6	15.73 (0.90)	15.44 (14.97-17.33)	
*Pde10a*	Right Cortex	Control	6	10.20 (1.84)	9.40 (8.83-13.60)	0.18
		BPA		9.36 (1.31)	8.86 (8.55-12.01)	
	Midbrain	Control	6	12.56 (0.78)	12.66 (11.36-13.60)	0.13
		BPA		11.91 (1.66)	11.56 (10.26-15.08)	
*Ppp1r9a*	Right Cortex	Control	6	7.601 (0.32)	7.587 (7.15-8.13)	0.58
		BPA		7.749 (0.52)	7.944 (7.06-8.24)	
	Midbrain	Control	6	8.753 (0.77)	9.783 (7.57-9.86)	0.93
		BPA		8.726 (0.61)	8.794 (8.01-9.62)	
*Dnmt1*	Right Cortex	Control	6	10.374 (0.29)	10.442 (9.88-10.63)	0.01
		BPA		9.471 (0.65)	9.454 (8.65-10.46)	
	Midbrain	Control	6	10.334 (0.42)	10.299 (9.66-10.78)	0.39
		BPA		9.895 (0.60)	10.008 (8.84-10.53)	
*Tet2*	Right Cortex	Control	6	13.867 (0.39)	14.055 (13.32-14.19)	0.13
		BPA		13.111 (1.42)	12.888 (11.46-15.54)	
	Midbrain	Control	6	14.195 (0.63)	14.427 (13.08-14.77)	0.03
		BPA		12.912 (0.65)	13.166 (12.09-13.53)	

a Mean and median values are the Ct values for each gene after normalization to housekeeping gene expression (delta Ct).

### Effect of BPA Exposure on Expression of Epigenetic Writers

**Figure 3:**
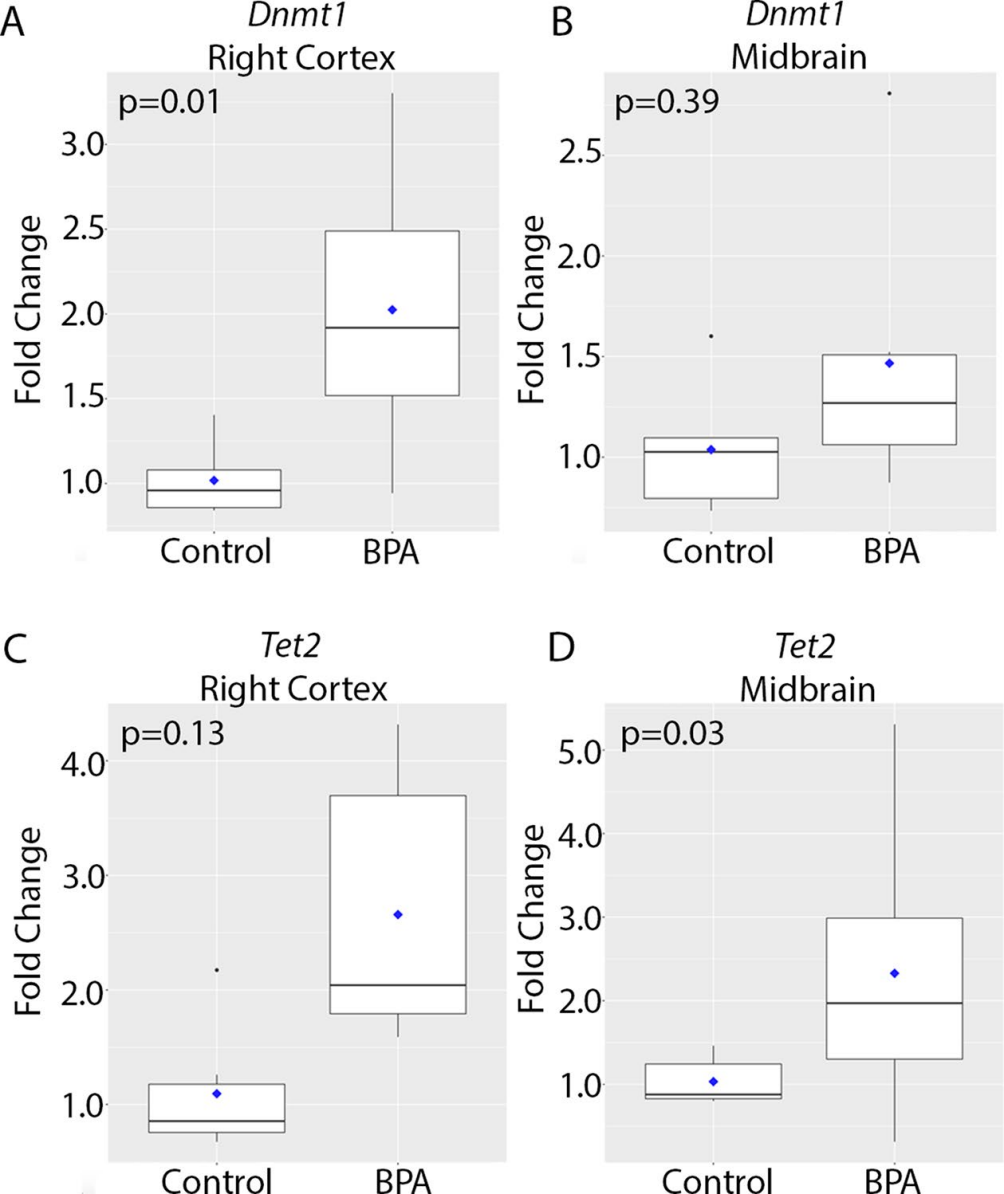
Expression of genes encoding epigenetic modifying enzymes in the brain at 10 months of age in animals exposed to control vs. BPA. Box plots depicting qRT-PCR data for *Dnmt1* **(A**, **B)** and *Tet2* **(C**, **D)** in the right cortex **(A**, **C)** and midbrain **(B**, **D)**. Data were analyzed using a two-sided non-parametric Wilcoxon test. Graphs depict the fold change in gene expression relative to the average of the control samples. n = 6 mice per exposure.

We next investigated whether epigenetic mechanisms contributed to the observed increase in *Kcnq1* expression in the brains of BPA-exposed animals. The conversion of cytosine to 5mC is catalyzed by DNA methyltransferases, including Dnmt1, and further oxidation of 5mC to 5hmC is mediated by Tet methylcytosine dioxygenases, including Tet1 and Tet2 (Jones, 2012; Antunes et al., 2019). Therefore, we investigated expression of *Dnmt1*, *Tet1*, and *Tet2* using qRT-PCR. *Dnmt1* expression was significantly higher in the right cortex of BPA-exposed mice compared to the control group (p = 0.01, **Figure 3A** and **Table 1**). In the midbrain, *Dnmt1* expression did not significantly differ between control and BPA-exposed groups (p = 0.4, **Figure 3B** and **Table 1**). Developmental BPA exposure had no significant effect on *Tet1* expression in either the right cortex or midbrain (**Supplementary Figure 1** and **Supplementary Table 3**). However, *Tet2* exhibited a trend toward increased expression in the right cortex (p = 0.13, **Figure 3C** and **Table 1**), and was significantly higher in the midbrain of BPA-exposed mice compared to controls (p = 0.03, **Figure 3D** and **Table 1**). Given the increased expression of *Dnmt1* and *Tet2* with BPA exposure, and their roles in epigenetic regulation of gene expression, we next evaluated whether expression of *Dnmt1* and *Tet2* were correlated with *Kcnq1* expression. Concomitant with the observed increase in *Kcnq1* expression with BPA exposure, expression of *Tet2* exhibited a trend toward significant correlation in the right cortex (p = 0.08), and was significantly positively correlated with *Kcnq1* expression in the midbrain (**Supplementary Figures 2A, B**). *Dnmt1* and *Kcnq1* expression exhibited a trend toward significant correlation in the right cortex (p = 0.06), and were not significantly correlated in the midbrain (**Supplementary Figures 2C, D**). Together, these data suggest that developmental BPA exposure leads to reprogramming of *Dnmt1* and *Tet2* expression in a brain region-dependent manner, concomitant with increased expression of *Kcnq1*.

### Effect of BPA on 5mC and 5hmC at the *Kcnq1* Locus

**Figure 4:**
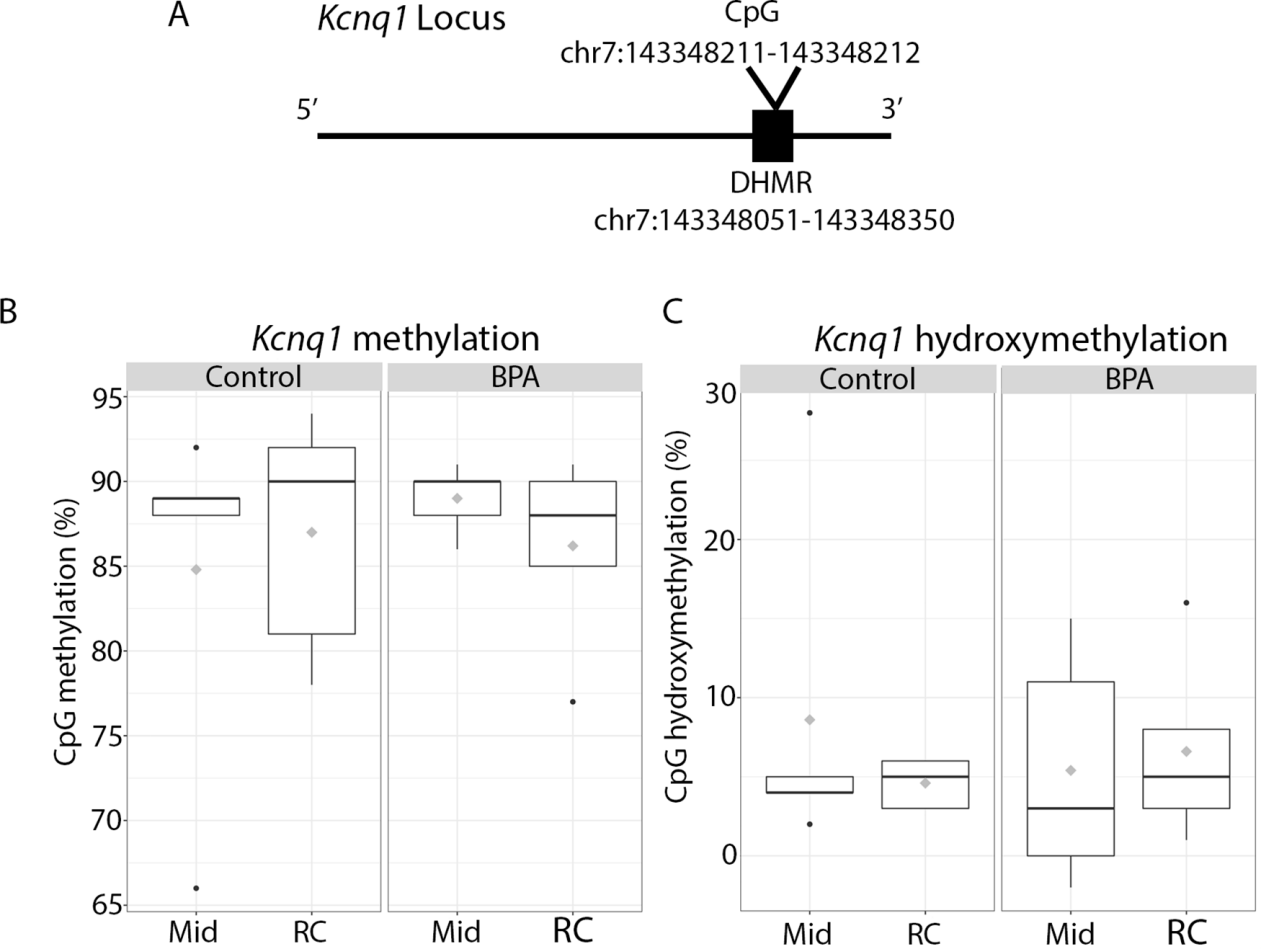
Enrichment of 5mC and 5hmC at the *Kcnq1* locus in 10 month old animals exposed to control vs. BPA. **(A)** Schematic of the *Kcnq1* locus, depicting the location of the CpG analyzed in this study. This CpG falls within an intronic region that was previously identified to be differentially hydroxymethylated with BPA exposure (DHMR) in the blood (J Kochmanski et al., 2018). **(B**, **C)** Box plots depicting CpG methylation **(B)** and hydroxymethylation **(C)** in the midbrain (Mid) and right cortex (RC) in control vs. exposed offspring. Raw pyrosequencing data can be found in **Supplementary Table 4**. Five samples from each exposure group/brain region passed the PyroMark quality control assessment and were used in the analysis. n = 5 mice per exposure/brain region.

Given the roles for Dnmt1 and Tet2 in regulation of 5mC and 5hmC, respectively, we next investigated whether perinatal BPA exposure resulted in altered 5mC and/or 5hmC at the *Kcnq1* locus. To address this question, we performed bisulfite conversion and oxidative bisulfite conversion of paired DNA samples from control and BPA-exposed right cortex and midbrain (see *Methods*). We chose a CpG site within an intronic region of the *Kcnq1* locus in which differential 5hmC was observed in blood samples of the same BPA-exposed mice (Kochmanski et al., 2018) (**Figure 4A**). Importantly, we discovered both 5mC and 5hmC at this CpG in both regions of the brain. The mean levels of 5mC in the right cortex were 87% and 86.2% in the control and BPA-exposed groups, respectively, and mean 5mC levels in the midbrain were 84.8% and 89% with control and BPA exposure, respectively (**Figure 4B** and **Table 2**). We observed no significant differences in 5mC with BPA exposure in either brain region (**Figure 4B** and **Table 2**). Levels of 5hmC at this locus were much lower than 5mC, consistent with literature demonstrating a lower level of 5hmC genome-wide compared to 5mC (Lopez et al., 2017). In the right cortex, mean 5hmC levels were 5.4% and 6.6% in the control and BPA-exposed group, respectively. In the midbrain, mean 5hmC levels were 8.6% and 4.6% in the control and BPA group, respectively (**Figure 4C** and **Table 2**). No significant differences in 5hmC levels were identified in either brain region with BPA exposure (**Figure 4C** and **Table 2**). We next investigated whether expression of *Dnmt1* and *Tet2* were associated with levels of 5mC and 5hmC at the *Kcnq1* locus. We observed no correlation between *Dnmt1* expression and levels of 5mC and 5hmC in the right cortex (**Supplementary Figure 3A**). However, we observed modest positive and negative correlations between *Dnmt1* expression and levels of 5mC and 5hmC, respectively, within the *Kcnq1* locus in the midbrain (**Supplementary Figure 3B**). No significant correlations were observed between *Tet1* or *Tet2* expression and 5mC/5hmC in either brain region (**Supplementary Figures 3C–F**). Finally, we determined whether *Kcnq1* expression correlated with levels of either 5mC or 5hmC at the *Kcnq1* locus. This analysis did not show significant correlation between *Kcnq1* expression and 5mC or 5hmC levels (**Supplementary Figures 4A, B**). These findings demonstrate enrichment with both 5mC and 5hmC at the *Kcnq1* locus, but suggest that BPA-induced changes in *Kcnq1, Dnmt1* and *Tet2* expression are not associated with altered 5mC or 5hmC at this site.

**Table 2:** DNA Methylation and Hydroxymethylation at *Kcnq1*.

Sample	Exposure	N^a^	% DNA methylation (SD)	P value (Mann-Whitney test)	Beta value^c^ (P Value)
5mC RC^b^	Control	5	87.0 (7.0)	0.6	1.2 (0.7)
	BPA	5	86.2 (5.6)		
5hmC RC	Control	5	5.4 (7.3)	0.6	
	BPA	5	6.6 (5.9)		
5mC Mid^b^	Control	5	84.8 (10.6)	0.8	-4.0 (0.4)
	BPA	5	89.0 (2.0)		
5hmC Mid	Control	5	8.6 (10.9)	0.9	
	BPA	5	4.6 (1.5)		

^a^Number of samples that passed pyrosequencing quality control and were included in statistical analysis. ^b^RC, Right Cortex, Mid, Midbrain. ^c^Beta value is the interaction term calculated using a linear mixed effects model. Methylation category (5mC or 5hmC), exposure group, and sex were included as terms in all models.

## Discussion

It is increasingly clear that exposures to endocrine disrupting chemicals, including BPA, during the perinatal period may interfere with normal programming of brain development and function (Patisaul, 2019). Normal development is orchestrated by epigenetic processes, and several studies have demonstrated that developmental BPA exposure results in disruption of the brain epigenome (Kundakovic et al., 2013; Kumar and Thakur, 2017; Drobna et al., 2018). In this work, we investigated the effects of perinatal BPA exposure on epigenetic regulation of imprinted genes in the brains of adult mice. We first examined expression of three imprinted genes, including *Kcnq1*, *Pde10a*, and *Ppp1r9a*. all of which have been reported to be expressed in the brain (Coskran et al., 2006; Goldman et al., 2009; Konopaske et al., 2015). We found that *Kcnq1* expression was significantly upregulated in the right cortex of 10 month old mice. To our knowledge, this is the first report demonstrating modulation of *Kcnq1* expression by developmental BPA exposure. *Kcnq1* encodes a voltage-gated potassium channel that is critical for normal cardiac repolarization, and it is also expressed in neurons (Goldman et al., 2009). Mutations in this channel are associated with seizures and fatal cardiac arrhythmias (Goldman et al., 2009). Moreover, recent evidence suggests that a single nucleotide polymorphism affecting *Kcnq1* expression in the brain is associated with schizophrenia spectrum disorder (Bruce et al., 2017). In light of this evidence and our findings, the functional consequences of increased *Kcnq1* expression in the brain as a result of environmental exposures warrant further investigation.

Programming of imprinted gene expression is governed by epigenetic mechanisms, particularly DNA methylation (Dolinoy et al., 2007). Therefore, we investigated the effects of perinatal BPA exposure on expression of *Dnmt1*, which encodes the DNA methyltransferase that is primarily responsible for maintenance of DNA methylation patterns across cell divisions (Jones, 2012). Recent evidence from our lab and others suggests a potential role for 5hmC in regulation of imprinted gene expression (Yamaguchi et al., 2013; Kochmanski et al., 2018). Therefore, we also investigated expression of the Tet methylcytosine dioxygenases *Tet1* and *Tet2*, which catalyze oxidation of 5mC to 5hmC (Antunes et al., 2019). *Dnmt1*, *Tet1*, and *Tet2* are expressed in neurons (Fan et al., 2001; Hsieh et al., 2016; Mi et al., 2015), and are also critical for differentiation of oligodendrocyte progenitors (Zhao et al., 2014; Moyon et al., 2017). Interestingly, expression of *Dnmt1* and *Tet2* were significantly increased in the right cortex and midbrain, respectively, of 10 month old animals, and exhibited trends toward increased expression in the other brain regions. These findings are consistent with research from others demonstrating changes in *Dnmt1* expression in the brain with perinatal BPA exposure (Kundakovic et al., 2013; Zhou et al., 2013; Kumar and Thakur, 2017). In contrast with the time point in later adulthood investigated in this work, these studies identified BPA-induced changes in *Dnmt1* expression at much earlier time points. The striking consistency of this finding by multiple research groups across several time points, and with varying experimental parameters (i.e. dose, method of administration of BPA), suggests that the effects of BPA on *Dnmt1* expression in the brain are robust. Prior to this study, the effects of perinatal BPA exposure on Tet enzyme expression had not yet been examined. However, low dose BPA exposure results in altered subcellular localization of Tet2 in hypothalamic neurons (Kurian et al., 2016), suggesting that BPA may target this enzyme *via* multiple mechanisms. Mutations and altered expression of *Dnmt1* and *Tet2* are associated with age-related cognitive decline and neurological diseases (Baets et al., 2015; Cui and Xu, 2018; Gontier et al., 2018). The effects of BPA on brain Dnmt and Tet enzyme levels across the lifecourse, and the health-related implications these changes, represent an important area for future studies.

The relationships between levels of 5mC/5hmC and gene expression are nuanced and dependent upon the genomic context (Jones, 2012). Methylation of CpGs at gene promoters is associated with repression of gene expression (Jones, 2012). In contrast, enrichment with 5hmC is generally associated with gene activation during development and in the adult brain (Ficz et al., 2011; Lin et al., 2017). Given the role for Dnmt and Tet proteins in regulation of imprinted gene expression (Barlow and Bartolomei, 2014; Liu et al., 2015), we investigated whether the expression of *Tet2* and *Dnmt1* were correlated with *Kcnq1* expression. In keeping with the function of Tet enzymes in gene activation, we observed a significant positive correlation between *Tet2* and *Kcnq1* expression in the midbrain. Indeed, we observed enrichment with 5hmC at the *Kcnq1* locus in the brain, an observation that has not yet been reported. However, no significant correlations were observed between expression of *Dnmt1* or *Tet2* and 5mC/5hmC at the *Kcnq1* locus. Moreover, there were no significant correlations between the levels of 5mC and 5hmC at the *Kcnq1* locus and expression of *Kcnq1*. Thus, given the small sample size, we were unable to determine potential mechanistic links between BPA exposure, alterations in 5mC and 5hmC at the *Kcnq1* locus, and increased *Kcnq1* expression. Further studies with larger sample sizes and assessment of additional CpGs in the *Kcnq1* locus are necessary to address this question. The observation of increased expression of *Kcnq1*, *Dnmt1* and *Tet2* with BPA exposure is notable, given that BPA exposure ceased at 3 weeks of age. The mechanism by which developmental BPA exposure mediates long-term programming of gene expression is unclear. However, recent evidence suggests that BPA can induce activation of the histone methyltransferase, MLL1, resulting in long-term programming of histone methylation (Wang et al., 2016). Whether a similar mechanism is responsible for our observations remains to be determined.

There are several limitations to this study. First, with six animals per exposure group, the sample size was small. Due to the variability inherent in *in vivo* studies, we were unable to ascertain statistically significant changes in 5mC and 5hmC with BPA exposure. In addition to increasing sample size, interrogation of additional CpGs in the *Kcnq1* locus may reveal further mechanistic insight into BPA-induced changes in *Kcnq1* expression. Moreover, although each exposure group contained three males and three females per group, the experiments were not adequately powered to look at sex-specific effects. Basal patterns of 5mC and 5hmC, and the effects of endocrine disruptors, including BPA, are highly sex-specific (Spiers et al., 2015; McCabe et al., 2017; Spiers et al., 2017). Indeed, sex-specific effects of BPA on *Dnmt1* expression have been reported (Kundakovic et al., 2013; Zhou et al., 2013; Wright et al., 2017). Thus, future studies controlling for sex may yield important biological insight, as well as potentially reduce variability in the data. Finally, it is increasingly clear that there is significant tissue and cellular heterogeneity with respect to gene expression and patterns of epigenetic marks (Cheow et al., 2016; Cusanovich et al., 2018). As bulk right cortex and midbrain were used in this study, the effects of BPA exposure on specific cellular populations in the brain are unclear. Nevertheless, in spite of these limitations, this is the first report to demonstrate that perinatal BPA exposure alters expression of *Kcnq1* and *Tet2* in the adult mouse brain, long after cessation of exposure. Moreover, consistent with previous studies, we provide further evidence that Dnmt1 is a target of BPA exposure. Given the critical roles for Dnmt1 and Tet2 in programming 5mC/5hmC during development and in adulthood, alterations in the expression of these enzymes may result in genome-wide epigenetic changes. The potential health implications of this programming by BPA exposure should be further investigated.

## Data Availability Statement

All datasets generated/analyzed for this study are included in the manuscript/**Supplementary files**.

## Ethics Statement

The animal study was reviewed and approved by University of Michigan Institutional Animal Care and Use Committee (IACUC).

## Author Contributions

MM, JK, LS, JG, JC, and DD planned experiments and assisted with troubleshooting. MM, JK, and TJ performed experiments. MM, JC, JG, DD, and LS analyzed data. MM and LS wrote manuscript.

## Funding

This work was supported by the Rackham Graduate Student Research Grant, University of Michigan, the UM NIEHS/EPA Children’s Environmental Health and Disease Prevention Center P01 ES022844/RD83543601, the Michigan Lifestage Environmental Exposures and Disease (M-LEEaD) NIEHS Core Center (P30 ES017885), and the UM NIEHS Institutional Training Grant T32 ES007062.

## Conflict of Interest

The authors declare that the research was conducted in the absence of any commercial or financial relationships that could be construed as a potential conflict of interest.
