# Supplementary material for: Perinatal Bisphenol A Exposure and Reprogramming of Imprinted Gene Expression in the Adult Mouse Brain
Source: Front Genet. 2019 Oct 10;10:951. doi: 10.3389/fgene.2019.00951 (PMC6796247; doi:10.3389/fgene.2019.00951)
Supplement: Supplementary file 2 [file Presentation_1.pdf]

Supplementary Table 1: qRT-PCR primer sequences

Gene	Forward	Reverse
Kcnq1	CATCACCCATGTGTCACAGC	CCGTGCTTGCTGGAATTTCT
Pde10a	CGGGACATCGAGCTATTCCA	ACTGCATGCTTCCAGTTGTG
Ppp1r9a	GCGGGCCTATTCTTCCTAGT	GCTGCTTGAAGCTTGGTCTT
Dnmt1	AGAGACGAGGATGAGAGGGA	GGTGAGTTGATCTTCGGGAT
Tet1	ACCGTGGTGTGTACGTTGAT	GTTCATCCTCGGGACAATTT
Tet2	GACTGCTGTTTGGGTCTGAA	GGTGACCACCACTGTACTGC
β-actin	GCTCCTTCGTTGCCGGTCCA	ATACAGCCCGGGGAGCATCG
18S	GCCCTGTAATTGGAATGAGTCCACTT	GTCCCCAAGATCCAACTACGAGCTTT
Gapdh	GCCTGCTTCACCACCTTCTT	CATGGCCTTCCGTGTTCCTA

Supplementary Table 2: Primer sequences for *Kcnq1* pyrosequencing

Primer	Sequence
Forward PCR	TGGGAGTTGAGTATATTTAGATAGGTA
Reverse PCR	AAACCATTTAACAAAAACTATCCCTCAACA
Sequencing	AAAACTATCCCTCAACAA

Supplementary Table 3: *Tet1* Summary Statistics

		N	Mean (sd)	Median (range)	Mann-Whitney test (α=0.05)
**Right Cortex**	Control	6	16.319 (1.39)	15.951 (15.252-19.055)	0.94
	BPA	6	15.759 (0.772)	15.759 (14.498-16.389)	
**Midbrain**	Control	6	15.824 (1.286)	16.037 (13.902-17.229)	0.31
	BPA	6	15.17 (0.796)	15.357 (13.803-15.912)	

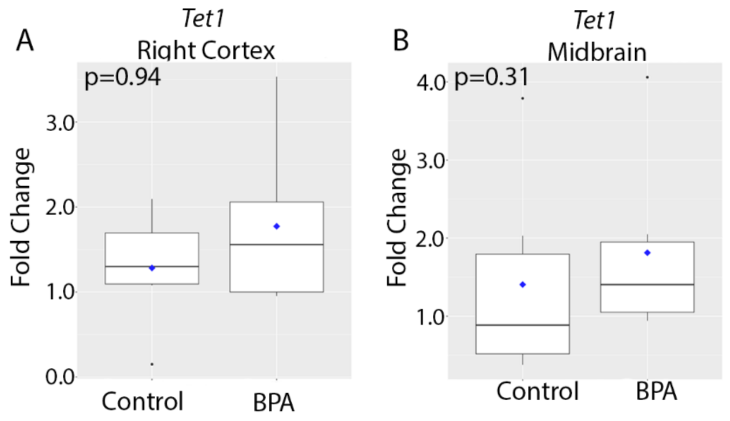

Supplementary Figure 1: *Tet1* expression in the brain at 10 months of age in animals exposed to control vs. BPA. Box plots depicting qRT-PCR data for *Tet1* in the right cortex (A) and midbrain (B). Data were analyzed using a two-sided non-parametric Wilcoxon test.

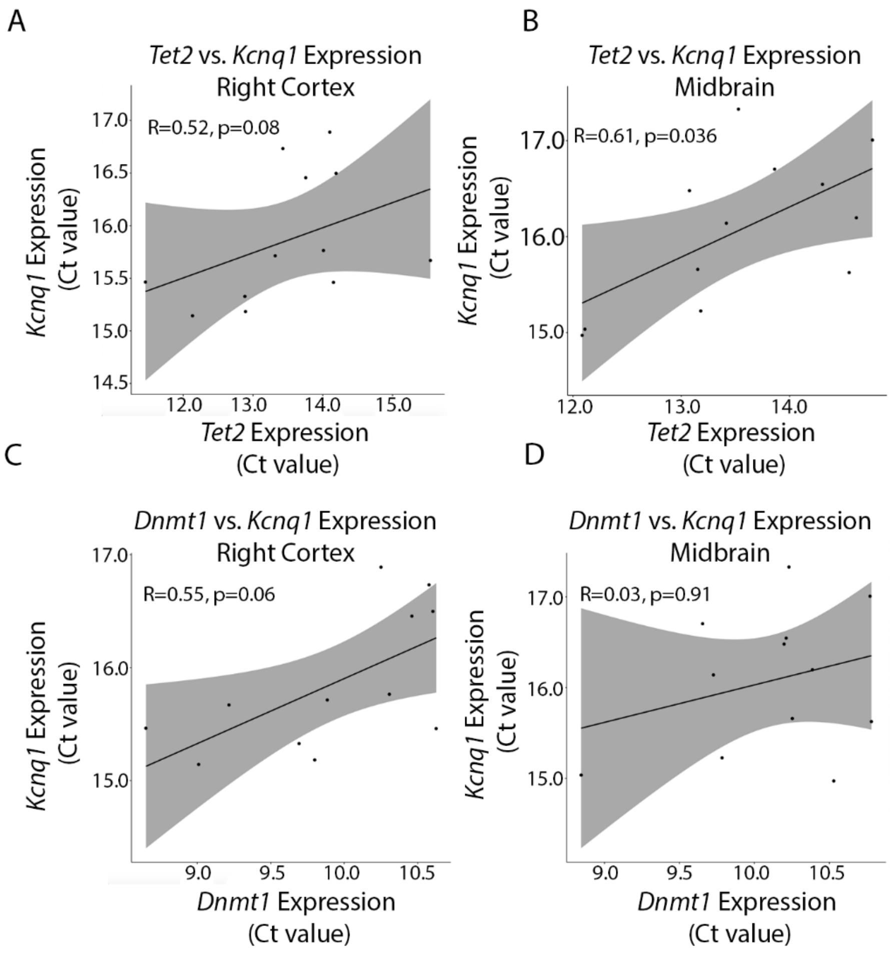

Supplementary Figure 2: Correlations between expression of *Tet2* (A-B) and *Dnmt1* (C-D) and *Kcnq1* expression in the right cortex (A,C) and midbrain (B,D). Correlation analyses were assessed with a Spearman Correlation Test using the ggpubr package in R. Ct values have been normalized to the average Ct value of the three housekeeping genes.

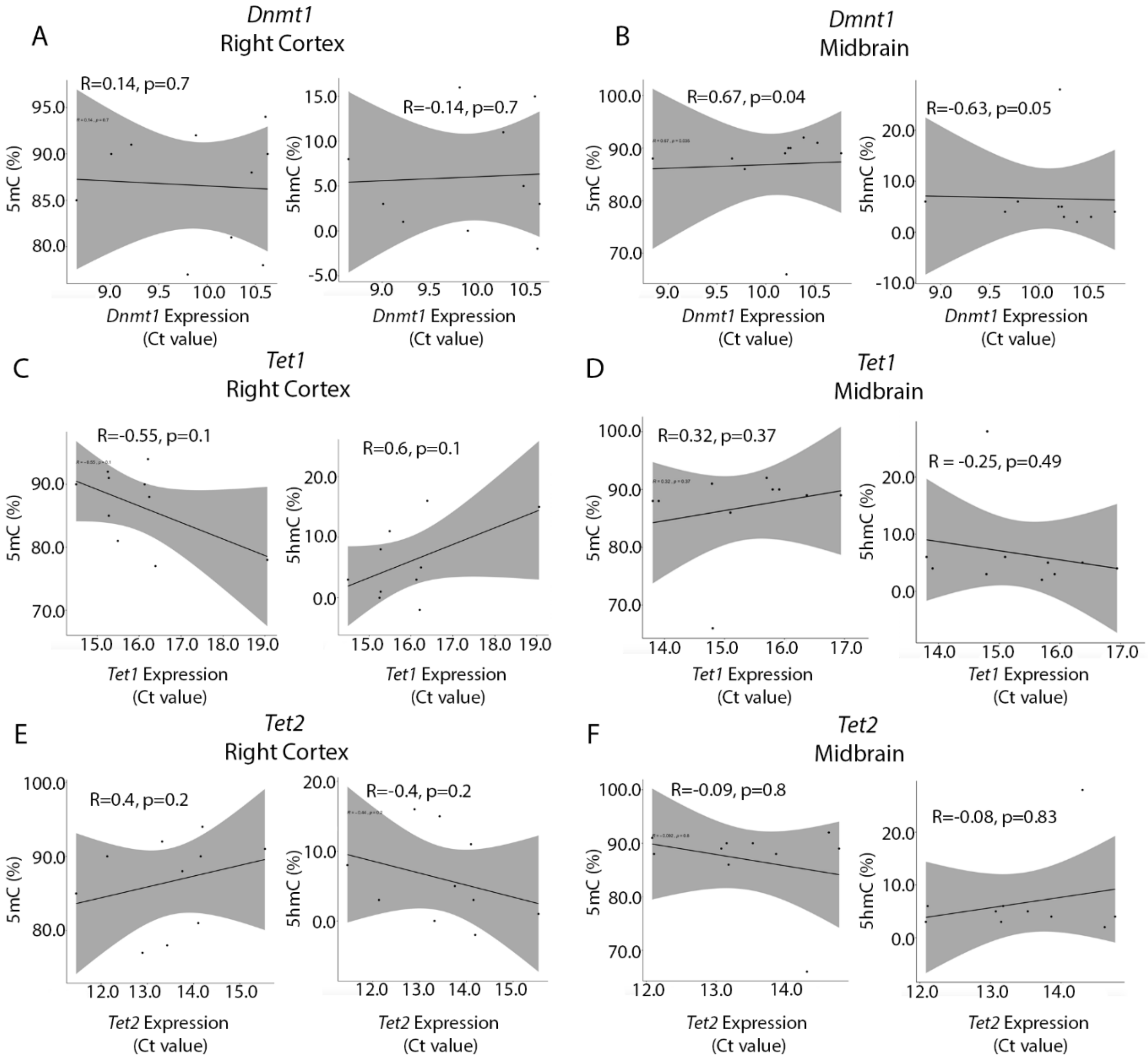

Supplementary Figure 3: Correlations between expression of *Dnmt1* (A-B), *Tet1* (C-D), *Tet2* (E-F) and levels of 5mC (left) and 5hmC (right). Correlation analyses were assessed with a Spearman Correlation Test using the ggpubr package in R. Ct values have been normalized to the average Ct value of the three housekeeping genes.

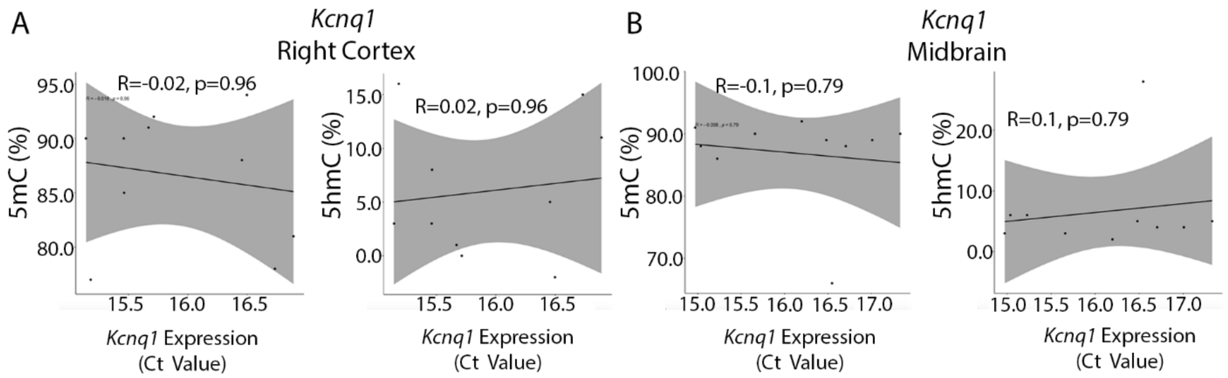

Supplementary Figure 4: Correlations between expression of *Kcnq1* and levels of 5mC (left) and 5hmC (right) in the right cortex (A) and midbrain (B). Correlation analyses were assessed with a Spearman Correlation Test using the ggpubr package in R. Ct values have been normalized to the average Ct value of the three housekeeping genes.
